# Canadian Children from Food Insecure Households Experience Low Self-Esteem and Self-Efficacy for Healthy Lifestyle Choices

**DOI:** 10.3390/nu11030675

**Published:** 2019-03-21

**Authors:** Stephanie L. Godrich, Olivia K. Loewen, Rosanne Blanchet, Noreen Willows, Paul Veugelers

**Affiliations:** 1 School of Medical and Health Science, Edith Cowan University, Bunbury, Western Australia 6230, Australia; 2 School of Public Health, University of Alberta, Edmonton, AB T6G 2P5, Canada; oloewen@ualberta.ca; 3 Department of Agricultural, Food & Nutritional Science, University of Alberta, Edmonton, AB T6G 2P5, Canada; rosanne.blanchet@ualberta.ca (R.B.); nwillows@ualberta.ca (N.W.)

**Keywords:** food security, self-esteem, self-efficacy

## Abstract

The objectives of this cross-sectional study were to: (i) determine whether there are differences in self-esteem and self-efficacy for healthy lifestyle choices between children living in food secure and food insecure households; and (ii) determine whether the association between household food insecurity (HFI), self-esteem and self-efficacy differs by gender. Survey responses of 5281 fifth-grade students (10 and 11 years of age) participating in the Canadian Children’s Lifestyle and School Performance Study II were analyzed using logistic and linear regression. HFI status was determined by the six-item short-form Household Food Security Survey Module (HFSSM). Students from food insecure households had significantly higher odds of low self-esteem, and significantly lower scores for global self-efficacy to make healthy choices, compared to students from food secure households. These associations were stronger for girls than for boys and appeared independent of parental educational attainment. Household income appeared to be the essential underlying determinant of the associations of food insecurity with self-esteem and self-efficacy. Upstream social policies such as improving the household income of low-income residents will reduce food insecurity and potentially improve self-esteem and self-efficacy for healthy choices among children. This may improve health and learning, and in the long term, job opportunities and household earnings.

## 1. Introduction

In Canada and the United States, household food insecurity (HFI) as measured on national surveys refers to self-reports of uncertain or insufficient food access, due to limited financial resources. Recent national prevalence data of HFI in Canada and the United States were reported as 12% [1] and 11.8% [2], respectively. These two countries use a validated tool, the 18-question Household Food Security Survey Module (HFSSM), to capture a gradient of deprivation within households. This ranges from anxiety about running out of food, to impacts on diet quality and quantity [2,3], which can have cognitive and physical health consequences [4].

Such consequences resulting from HFI include multiple poor health outcomes, higher health care utilization and costs [5,6], higher mortality rates [7] and adverse mental health outcomes such as behavioural issues, distress, anxiety, depression and suicidal thoughts [6,8,9,10]. Furthermore, HFI has a graded negative effect on a variety of physical and mental health outcomes, in which more severe food insecurity (FI) is associated with increased risk of adverse outcomes [10]. Among children, HFI has been associated with impaired mental health [11], reduced academic performance [12], absenteeism [13], and poorer behavioural outcomes [12,14]. HFI may affect girls and boys differently and associations between HFI, children’s academic performance, social skills and feelings of fear have been shown to differ between gender, with the effect more pronounced for girls [15,16]. HFI seems to influence children’s development through its effects as a component of overall family stress and poor functioning [12,17,18]. The evidence thus indicates that HFI is a critical public health issue that merits attention, given its importance in determining short and long-term health outcomes among children and adults [19,20].

As HFI experienced in childhood is a stressor and precursor to unfavourable cognitive outcomes in childhood and adulthood [12], it is important to build self-confidence early in life. Self-esteem can be described as the attitude towards oneself [21], which can be positive or negative [22]. Self-efficacy is an individual’s belief in their ability to achieve goals [23], such as healthy eating (HE) or physical activity (PA) goals. People with high self-efficacy have higher confidence in their ability to translate intentions into behaviours [24]. With frequent practice, self-efficacy relating to healthy behaviour goals, such as meal preparation in the home environment, can be increased [25]. High self-efficacy has been shown in adult populations to increase diet quality [26]. The limited existing literature also suggests an association between FI and self-efficacy [27]. Self-efficacy seems to be impaired by FI [28] and improvements in food security (FS)—regular and reliable access to sufficient nutritious food [29]—have been shown to increase self-efficacy [30]. This may in turn enhance academic achievement and “economic productivity” [12]. Given that low self-esteem and self-efficacy in childhood may be an “enduring vulnerability” for mental health issues in adulthood [31,32], it is important to ensure positive self-esteem and self-efficacy are fostered in childhood [33]. This is particularly important among girls, who will become mothers, given HFI risk is increased by maternal depression [34] and food insecure mothers are more likely to report their own experiences of childhood deprivation [35]. Therefore, addressing FI during childhood could reduce the likelihood of intergenerational FI [35].

In order to prevent mental health issues, greater understanding of how FI influences self-esteem and self-efficacy among children is required. Further, the limited evidence of the association between household FS status and a child’s perceived self-efficacy to make healthy lifestyle choices, such as PA and HE needs to be extended. Therefore, the aim of this paper is to examine the relationship between household FS and self-esteem and self-efficacy for healthy lifestyle choices among grade five children living in Nova Scotia, Canada, where 22.8% of children lived in FI households in 2015–16 [36]. The relationship is examined by classifying HFI using the six-item short form HFSSM into three levels (marginal, moderate and severe). The objectives included: (i) to determine whether there are differences in self-esteem and self-efficacy for healthy lifestyle choices between students living in households with FS and FI; and (ii) to determine whether there are gender differences within the association between FI, self-esteem and self-efficacy among children. We hypothesized that students from households experiencing FI would have lower self-esteem and lower self-efficacy for HE and participating in PA, and that this association would differ between genders.

## 2. Materials and Methods

### 2.1. Sampling and Recruitment

In this cross-sectional study, we used data from the Children’s Lifestyle and School Performance Study II (CLASS II) conducted in 2011 in Nova Scotia, Canada. Nova Scotia is a province on the eastern coast of Canada, consisting of a peninsula and offshore islands with a population just under 1 million people [37]. CLASS II was a population-based survey that examined diet, physical activity, well-being, and school performance among fifth-grade students (10–11 years old). All grade five students in the province, their parent(s)/guardian(s), and school administrators were invited to participate in the study. Of all 286 provincial public schools with grade five students, 269 schools (94.1%) participated in the study. Once a school agreed to participate, parents or guardians received a home package containing a consent form and survey to complete. Parental consent to participate in the survey was given for 6591 of the 8736 students (75.4% consent rate). Of these, 1310 (19.9%) students did not complete the survey, were absent the day of the survey, or had returned an incomplete parent survey and were excluded from analysis, leaving 5281 eligible students. Of these, 5093 (96.4%) and 5113 (96.8%) had complete data for measures of self-esteem and self-efficacy items, respectively. 

### 2.2. Instrument

The student survey consisted of questions on eating behaviours at school and home, physical activity, mental wellbeing, self-esteem, and self-efficacy. Parent(s)/guardian(s)’ survey contained questions about the home environment, household FI experience, and sociodemographic factors. Specifically, the home survey contained the US Department of Agriculture 6-item short-form Household Food Security Survey Module (HFSSM), a tool that has been validated in the US and is recommended when there is the need to reduce respondent burden or when asking questions about children’ FI is deemed too sensitive [3]. A score was calculated from the number of affirmative responses to the six questions about FI. Based on overall score, households were classified as food secure (score 0), marginal FI (score 1), moderate FI (score 2–4) or severe FI (5–6) [14]. FI status constituted the exposure of interest. 

Outcomes included global self-esteem, assessed using a series of ten questions with a 3-point Likert scale response options similar to the Emotional Functioning and Social Functioning items on the PedsQL [38]. The following items were included (i) My future looks good to me; (ii) I like the way I look; (iii) I like myself; (iv) I feel like I do not have any friends; (v) I feel unhappy or sad; (vi) I worry a lot; (vii) I am in trouble with my teacher(s); (viii) I have trouble paying attention; (ix) I have trouble enjoying myself; (x) If I have problems there is someone I trust to go to for advice. Response choices for each of these items included ‘never or almost never’, ‘sometimes’, to ‘often or almost always’. The responses were scored as 1, 2, 3, with the highest score representing good self-esteem. Scores were subsequently totaled. The inter-item reliability (Cronbach’s alpha) of the 10 items was 0.70.

Outcomes also included self-efficacy for healthy lifestyle choices, assessed using 9 items, 4 for physical activity and 5 for healthy eating. Students were asked “If you wanted to, how confident are you that you could (i) be physically active no matter how tired you may be?; (ii) be physically active even if you have a lot of homework?; (iii) ask your parent or other adult to play a physical activity or sport with you?; (iv) be physically active for at least 60 min on 5 or more days per week?; (v) eat healthy food at school?; (vi) choose a healthy snack between school and dinner time?; (vii) eat healthy food if you are alone at home?; (viii) choose a healthy snack when you are bored?; (ix) choose a healthy snack when you are sad?” Response choices included ‘not at all confident’, ‘a little bit confident’, ‘quite confident’, and ‘very confident’. The responses were scored as 1, 2, 3, 4, with the highest score representing higher self-efficacy. The scores were summed for global self-efficacy (all nine items), physical activity, and healthy eating. These scores were then transformed to a scale of 1 to 100 to make them comparable across the three measures on self-efficacy. The items for self-efficacy have each demonstrated good internal consistency [39]. The inter-item reliability (Cronbach’s alpha) of the physical activity, healthy eating self-efficacy, and global self-efficacy items were 0.69, 0.83, and 0.83 respectively.

Covariates used in analyses included gender, region of residence (urban or rural; based on postal code), parental educational attainment, household income, and bodyweight status [40] (normal weight, overweight, or obese using age and gender specific cut-offs). Further information about the CLASS study can be found at www.nsclass.ca.

### 2.3. Data Collection

The student survey was pilot tested for ease of understanding and reliability. Trained CLASS research assistants travelled to participating schools and administered surveys to students who had returned a signed consent to participate. Research assistants measured students’ height to the nearest 0.1 cm and weight to the nearest 0.1 kg on calibrated digital scales. Additional data collection information has been detailed elsewhere [41].

### 2.4. Data Entry and Analysis

We conducted descriptive analysis of associations between FI status, gender, region of residence, parent education, household income, and bodyweight status with self-esteem and self-efficacy using Chi^2^ tests. For the descriptive analyses, self-efficacy scores were split into tertiles.

For self-esteem items, the responses were ordinal and the sum scores were not normally distributed. Self-esteem was therefore dichotomized whereby sum scores lower than the 15th percentile were defined as low self-esteem, which is similar to the parametric concept of one standard deviation below the mean, an approach commonly applied in self-esteem research [42]. Univariate logistic regression was used to examine the association of FI status and confounders with the dichotomized self-esteem scores. A multivariable mixed-effects logistic regression with robust standard errors was used to account for clustering of students within schools and the confounding potential of gender, region of residence, and bodyweight status. Later multivariable models were sequentially further adjusted for parent education and household income. In addition, these models were tested for gender-HFI interaction and found to be significant (*p* = 0.006). As such, gender-stratified multivariable models, adjusted for the same covariates (except gender), were used to examine whether the associations were distinct for girls and boys.

For the associations of self-efficacy with FI status, univariate linear regression models were conducted for global, PA, and HE self-efficacy scores. A multivariable linear regression model with robust standard errors was used to account for clustering of students and adjusted for gender, region of residence, and bodyweight status. This model was further adjusted for parental education and household income. Models were tested for gender-HFI interaction, but it was not found to be significant (*p* = 0.251). For consistency with self-esteem models, gender-stratified analyses was conducted using a multivariable model, adjusting for the same covariates (except gender).

All analyses were weighted to represent provincial estimates of the grade 5 student population in Nova Scotia. Responses rates in residential areas with lower household income were slightly lower than average and weights were calculated to account for this disproportionate non-response [43]. Missing values for potential confounders were considered as separate covariate categories, but their estimates are not presented. Normality and homoscedasticity were tested and found to be acceptable for all linear regression models. All analyses were conducted using the statistical software package Stata/IC 14 and *p* < 0.05 were considered significant.

The Health Sciences and Human Research Ethics Board of Dalhousie University approved the original study, including the informed consent procedure. The Health Research Ethics Board at the University of Alberta approved the data analysis of the present study. Edith Cowan University Human Research Ethics Committee provided multicentre research project approval.

## 3. Results

### 3.1. Participant Demographics

Table 1 presents characteristics of grade five students in Nova Scotia and shows that 52.2% were girls, 35.2% resided in rural regions, and 54.3%, 22.7%, and 17.8% had normal weight, overweight, or obesity, respectively.

### 3.2. Household Food Security Status

As shown in Table 1, 74.8% of students were from households classified as food secure, 8.1% from households with marginal FI, 10.0% from households with moderate FI, and 7.1% from households with severe FI, for a combined prevalence of 25.2% HFI.

### 3.3. Global Self-Esteem and Self-Efficacy for Healthy Lifestyle Choices

Of students with low self-esteem, 9.4% were from households experiencing severe FI whereas among students with normal self-esteem only 6.6% came from FI households. Likewise, among students in the lowest self-efficacy tertile, a higher percentage (8.3%) were from severe FI households relative to students in the highest self-efficacy tertile (5.7%). These differences were statistically significant (*p*’s ≤ 0.001). Similar statistically significant gradients were observed for household income and parental education, with lower household income and parental education associated with lower self-esteem and self-efficacy (data not shown).

### 3.4. The Association between Household Food Security and Self-Esteem

Table 2 presents the univariate and multivariable associations between HFI status and low self-esteem for students. Students (girls and boys combined) from households with marginal, moderate, or severe FI had respectively 44%, 55%, and 54% higher odds of having low self-esteem compared to students from food secure households after adjusting for gender, region of residence and bodyweight. The interaction between FI and gender was found to be statistically significant (*p* = 0.006). In the gender-stratified Model 1, the association with lower self-esteem was only significant for girls from moderate FI households and boys from households with severe FI (girls: OR 2.19; boys: OR: 1.68, compared with their FS counterpart). This association was not significant among girls living in marginal or severe FI households or among boys living in marginal FI or moderate FI. When this model was further adjusted for parent educational attainment (Model 2), the association between HFI and low self-esteem remained significant only among students (girls and boys combined) and girls living in moderate FI households. They had respectively 40% and 2 times higher odds of low self-esteem when compared to their FS counterpart. Model 3 was further adjusted for household income. It showed that girls from moderate FI households had an associated 67% higher odds of low self-esteem when compared to their FS counterpart. This association was no longer significant for girls and boys combined.

### 3.5. The Association between Household Food Security Status and Self-Efficacy

Table 3 presents the associations between FI and self-efficacy. Students from moderate and severe FI households had significantly lower scores for global self-efficacy to make healthy choices than students from food secure households. The interaction between FI and gender in their association with self-efficacy was not statistically significant (*p* = 0.251). The associations of FI with global self-efficacy score remained statistically significant after adjusting for gender, bodyweight status, and region of residence, while they remained statistically significant only for girls in the gender-stratified Model 1. When these models were further adjusted for parent educational attainment, associations remained significant only for students (girls and boys combined) and for girls from moderate FI households (Model 2). The association between FI and global self-efficacy was no longer significant after further adjusting for household income.

The individual associations of FI with self-efficacy for physical activity and healthy eating are presented in the Supplementary Table S1. With respect to self-efficacy for physical activity, students from households with moderate and severe FI had significantly lower scores compared to students from food secure households. No differences were observed when comparing children from households with marginal FI and with FS. Associations remained after adjusting for gender, bodyweight status, and region of residence. In the gender-stratified models, this association remained only for girls. With the exception of girls from moderately FI households having significantly lower associated self-efficacy for physical activity after adjusting for parental education, associations did not remain statistically significant after further adjusting for parent education and household income.

In terms of self-efficacy for healthy eating, students from moderately FI households had significantly lower scores than students from food secure households before and after adjusting for gender, bodyweight status, and region of residence. In gender-stratified models, this difference was significant for girls from moderate and severe FI households, but not for boys. The association with lower self-efficacy for healthy eating remained significant only among students (girls and boys combined) and girls living in moderate FI households after further considering parental education. When this model was further adjusted for household income, the association remained significant only for girls from moderately FI households (Supplementary Table S1).

## 4. Discussion

The objectives of this study were to: (i) to determine whether there are differences in self-esteem and self-efficacy for healthy lifestyle choices between students living in households with FS and FI; and (ii) to determine whether there are gender differences within the association between FI, self-esteem and self-efficacy among children. Our findings revealed that one quarter (25.2%) of children who participated in the CLASS II study were living in food insecure households. We demonstrated that these children living in households with FI were more likely to have low global self-esteem and low self-efficacy for healthy lifestyle choices. These associations were generally more pronounced for girls than for boys and independent of region of residence, bodyweight status and parental educational attainment. Household income appeared to be the essential underlying determinant of the associations of FI with self-esteem and self-efficacy, to some extent lesser in the association in girls. Although 7.1% of children lived in households with severe FI, we did not observe a gradient of severity of HFI with likelihood of low self-esteem and self-efficacy for physical activity and healthy eating.

Our observed FI prevalence (25.2%) was relatively consistent with provincial estimates of HFI for the same year (2011) in that 23% of Nova Scotian households with children were food insecure [19]. Findings from the present study relating to FI, self-esteem and self-efficacy are supported by previous literature [30,44]. However, as existing research that has measured the association between FI and self-esteem among children is limited, we have positioned our findings amongst studies of adults. Laraia et al. (2006) reported a negative association among self-esteem and FI among women [44]. A similar inverse relationship between self-efficacy and FI was observed among adults in the United States; FI was associated with low levels of self-efficacy [27]. Martin et al. (2016) investigated whether an association existed between self-efficacy and FS among adults. The authors found a significant inverse association between FI and self-efficacy [30]. Our findings corroborate these results.

### 4.1. Policy, Practice and Research Recommendations

Given we found self-esteem and self-efficacy were compromised among children from food insecure households, especially among girls, upstream policy actions targeting the structural determinants of HFI [45] should be prioritized. Our research is suggestive that such action would not only reduce FI prevalence but could also prevent resultant implications for children’s mental health status and life chances, given that living in households with FI manifests in mental health issues in adolescence and adulthood [12,46,47]. As such, our recommendation to address HFI includes social policy interventions to increase the household income of low-income residents [48]. The relationship between income and HFI suggests increasing these residents’ income, such as through a Basic Income Guarantee, could have a substantial impact on FI [49]. Other important implications of this research include incorporating strategies to increase self-esteem and self-efficacy, such as for example in school settings [27] because improved lifestyle behaviours have also been related to better school achievement and reduced prevalence of mental health issues [50,51]. Further, promoting childhood self-esteem and self-efficacy for healthy lifestyle choices may improve health and learning, and herewith future job opportunities and earnings. Population groups that require targeted support include girls, given the current study suggests they are more vulnerable to low self-esteem and self-efficacy if they are living in food insecure households [47,52]. Additionally, there is a relationship between positive self-efficacy and mental wellbeing [32]. Therefore, strategies must be implemented to ensure the lasting trajectory towards both FI and negative psychological outcomes is mitigated during childhood [46].

Further research demonstrating the relationship between HFI and low self-esteem and self-efficacy is warranted, given that low self-esteem contributes to depression in youth [32,53]. This research should also be undertaken in other similar countries, for comparison purposes.

### 4.2. Strengths and Limitations

Strengths of this study include the large population-based sample, increasing the study’s representativeness. Sampling accounted for urban and rural residents, with all public schools in the province invited to participate. The relative high participation rate was another strength. To our knowledge, no other studies have investigated the gender differences in children between HFI modelled as three levels of severity and self-esteem and self-efficacy, reinforcing the importance of our contribution to the scarce evidence base. However, there were limitations associated with this study. This research was cross-sectional in nature, and therefore we could not establish causation. A review of longitudinal studies investigating associations between FI and mental health in adults suggested a bidirectional relationship existed (for example FI increasing depression and depression increasing FI) [54]. Therefore, low self-esteem may contribute to increasing FI over time in adults. However, research among children is insufficient to understand this relationship. A previous study has suggested that the cost of treating children’s mental health care may negatively impact household finances, thus resulting in HFI [55]. However, further research in the field is required and the cross-sectional nature of the present study precludes further investigation into the temporality of the association among our sample. Although responses rates were lower than average among residential areas with lower household income, weights were calculated to correct for this disproportionate non-response. The use of the 6-item HFSSM does not capture the most severe range of FI, nor does it capture anxiety or concerns with regards to accessing food [3]. In addition, it does not inquire about FI among children within the household, and thus, limits our ability to directly associate findings with child FI. However, evidence suggests that HFI independently predicts individuals’ health [56] and that living in an adult food insecure household is sufficient for children to suffer from consequences of FI [3]. Further, the six-item short-form is slightly less reliable and sensitive than the full 18-item questionnaire [3,57], and as such, the prevalence reported in our research may be underestimated. This shortcoming also has the potential to diminish the strength of association between HFI and self-esteem and self-efficacy. Lastly, though having demonstrated good internal reliability, the lack of standardized self-esteem and self-efficacy tools is a limitation of this research.

## 5. Conclusions

Various studies examined the association between HFI and poor mental health in high-income countries such as Canada. These studies generally concluded that HFI is associated with poor mental health outcomes. In this study, we examined in children if self-esteem and self-efficacy were associated with HFI at three increasing levels of severity. Given there is a hypothetical cyclical relationship between FI and self-esteem and self-efficacy, interventions that address both child poverty, low-self-esteem and low self-efficacy are required to break the cycle of intergenerational HFI. Such actions could improve physical and cognitive health among children now and throughout their entire lifespan. This is critically important, given the link between mental health problems in childhood and in later life [11]. 

## Figures and Tables

**Table 1:** Characteristics of grade 5 students (aged 10–11 years) in Nova Scotia, Canada by self-esteem and self-efficacy. Children’s Lifestyle and School performance Study (CLASS) 2011.

	Total Sample (*n* = 5281)	Self-Esteem (*n* = 5093)		Self-Efficacy (*n* = 5113)		
		Low	Normal	Lowest Tertile	Middle Tertile	Highest Tertile
**Household Food Security Status**						
Food secure	74.8%	66.8%	76.2%	72.2%	75.2%	77.5%
Marginal FI ^1^	8.1%	10.1%	7.7%	7.9%	8.3%	8.2%
Moderate FI ^1^	10.0%	13.7%	9.5%	11.6%	9.5%	8.6%
Severe FI ^1^	7.1%	9.4%	6.6%	8.3%	7.0%	5.7%
**Girls (%)**	52.2%	47.8%	52.9%	49.5%	54.2%	52.9%
**Bodyweight status (%)**						
Normal weight	54.3%	43.3%	56.0%	50.5%	53.5%	59.9%
Overweight	21.7%	22.3%	21.7%	21.5%	22.7%	21.2%
Obesity	17.8%	28.2%	16.3%	20.9%	18.1%	14.1%
Missing	6.1%	6.3%	6.0%	7.1%	5.8%	4.9%
**Rural Residence (%)**	35.2%	41.6%	34.4%	37.3%	36.1%	32.9%
**Household Income (%)**						
<$20,000	20.7%	31.9%	18.8%	23.9%	19.4%	17.5%
$20,001–40,000	14.1%	14.5%	14.0%	15.0%	14.2%	12.6%
$40,001–60,000	25.4%	21.8%	26.0%	24.3%	26.6%	26.3%
>$60,000	20.6%	12.9%	21.8%	16.3%	21.5%	24.7%
Missing/prefer not to answer	19.2%	18.8%	19.4%	20.5%	18.3%	18.9%
**Parent education (%)**						
Secondary school or less	18.0%	23.0%	17.2%	20.8%	16.5%	16.2%
College	40.6%	44.5%	39.9%	41.7%	43.3%	36.0%
University	37.5%	27.0%	39.3%	32.5%	37.2%	42.3%
Missing	4.0%	5.6%	3.7%	5.1%	3.0%	3.5%

^1^ Food insecurity.

**Table 2:** Relationship between food security and low self-esteem among grade 5 students (aged 10–11 years) in Nova Scotia, Canada.

Self-Esteem						
	All Students		Girls		Boys	
	OR (95%CI)	*p*-Value	OR (95%CI)	*p*-Value	OR (95%CI)	*p*-Value
	Univariate					
**Household Food Security Status**						
Food secure	1		1	.	1	.
Marginal FI ^1^	**1.49 (1.10, 2.03)**	**0.010**	**1.56 (1.05, 2.32)**	**0.028**	1.49 (0.93, 2.40)	0.095
Moderate FI ^1^	**1.65 (1.25, 2.18)**	**<0.001**	**2.39 (1.71, 3.33)**	**<0.001**	1.07 (0.69, 1.68)	0.753
Severe FI ^1^	**1.62 (1.19, 2.20)**	**0.002**	1.57 (0.97, 2.52)	0.066	**1.73 (1.16, 2.58)**	**0.007**
	**Model 1**					
Food secure	1		1	.	1	.
Marginal FI ^1^	**1.44 (1.05, 1.97)**	**0.022**	1.47 (0.96, 2.23)	0.073	1.46 (0.91, 2.34)	0.120
Moderate FI ^1^	**1.55 (1.18, 2.05)**	**0.002**	**2.19 (1.57, 3.07)**	**<0.001**	1.04 (0.66, 1.63)	0.869
Severe FI ^1^	**1.54 (1.12, 2.12)**	**0.009**	1.42 (0.87, 2.31)	0.158	**1.68 (1.11, 2.53)**	**0.014**
	**Model 2**					
Food secure	1		1	.	1	.
Marginal FI ^1^	1.35 (0.99, 1.85)	0.058	1.37 (0.90, 2.09)	0.146	1.38 (0.86, 2.20)	0.180
Moderate FI ^1^	**1.40 (1.06, 1.85)**	**0.020**	**2.00 (1.42, 2.81)**	**<0.001**	0.93 (0.59, 1.45)	0.740
Severe FI ^1^	1.35 (0.99, 1.85)	0.062	1.27 (0.77, 2.09)	0.352	1.46 (0.97, 2.19)	0.069
	**Model 3**					
Food secure	1		1	.	1	.
Marginal FI ^1^	1.17 (0.85, 1.60)	0.330	1.16 (0.75, 1.79)	0.503	1.19 (0.74, 1.91)	0.474
Moderate FI ^1^	1.16 (0.87, 1.55)	0.317	**1.67 (1.16, 2.41)**	**0.006**	0.77 (0.49, 1.23)	0.275
Severe FI ^1^	1.01 (0.73, 1.41)	0.930	0.99 (0.58, 1.68)	0.972	1.06 (0.68, 1.65)	0.788

OR: Odds Ratio; 95% CI; 95% confidence interval; Model 1 is adjusted for region of residence, body weight status, and gender (in non-gender-stratified models). Model 2 is further adjusted for parental education and Model 3 is further adjusted for household income. Estimates are weighted to represent grade five students in Nova Scotia. Results in bold are statistically significant (*p* < 0.05); ^1^ Food insecurity.

**Table 3:** Relationship between food insecurity and global self-efficacy to make healthy choices among grade 5 students (aged 10–11 years) in Nova Scotia, Canada.

Global Self-Efficacy						
	All Students		Girls		Boys	
	B (95%CI)	*p*-Value	B (95%CI)	*p*-Value	B (95%CI)	*p*-Value
	Univariate					
**Household Food Security Status**						
Food secure	0.00	-	0.00	-	0.00	-
Marginal FI ^1^	−1.03 (−2.69, 0.62)	0.221	−0.98 (−3.20, 1.23)	0.383	−1.32 (−3.55, 0.91)	0.244
Moderate FI ^1^	**−2.73 (−4.19, −1.27)**	**<0.001**	**−3.16 (−4.92, −1.40)**	**<0.001**	−2.29 (−4.57, 0.00)	0.05
Severe FI ^1^	**−2.27 (−4.04, −0.48)**	**0.013**	**−3.17 (−5.44, −0.90)**	**0.006**	−1.29 (−3.92, 1.33)	0.332
	**Model 1**					
Food secure	0.00	-	0.00	-	0.00	-
Marginal FI ^1^	−0.82 (−2.48, 0.84)	0.331	−0.67 (−2.89, 1.56)	0.555	−1.14 (−3.32, 1.05)	0.305
Moderate FI ^1^	**−2.41 (−3.83, −0.98)**	**0.001**	**−2.82 (−4.59, −1.04)**	**0.002**	−1.93 (−4.20, 0.33)	0.094
Severe FI ^1^	**−2.02 (−3.78, −0.25)**	**0.025**	**−2.83 (−5.06, −0.60)**	**0.013**	−0.99 (−3.65, 1.66)	0.461
	**Model 2**					
Food secure	0.00		0.00	-	0.00	-
Marginal FI ^1^	−0.31 (−1.97, 1.35)	0.710	−0.32 (−2.53, 1.89)	0.774	−0.51 (−2.71, 1.69)	0.646
Moderate FI ^1^	**−1.58 (−3.01, −0.15)**	**0.030**	**−2.37 (−4.13, −0.62)**	**0.008**	−0.66 (−2.96, 1.63)	0.571
Severe FI ^1^	−1.00 (−2.79, 0.79)	0.273	−2.24 (−4.48, 0.013)	0.051	0.52 (−2.12, 3.16)	0.699
	**Model 3**					
Food secure	0.00	-	0.00	-	0.00	
Marginal FI ^1^	0.24 (−1.41, 1.90)	0.774	0.27 (−1.92, 2.47)	0.806	0.10 (−2.16, 2.36)	0.930
Moderate FI ^1^	−0.90 (−2.38,0.58)	0.232	−1.80 (−3.61, 0.01)	0.052	0.084 (−2.33, 2.51)	0.946
Severe FI ^1^	−0.081 (−2.03, 1.87)	−0.935	−1.56 (−4.04, 0.91)	0.215	1.64 (−1.17, 4.44)	0.249

B: regression coefficient; 95% CI; 95% confidence interval; Model 1 is adjusted for region of residence, body weight status, and gender (in non-gender-stratified models). Model 2 is further adjusted for parental education and Model 3 is further adjusted for household income. Estimates are weighted to represent grade five students in Nova Scotia. Results in bold are statistically significant (*p* < 0.05); ^1^ Food insecurity.

## Supplementary Materials

The following are available online at https://www.mdpi.com/2072-6643/11/3/675/s1, Table S1: Relationship between food insecurity and self-efficacy for physical activity and healthy eating among grade 5 students (aged 10–11 years) in Nova Scotia, Canada.

## References

[1] Tarasuk V., Mitchell A., Dachner N. (2016). Household Food Insecurity in Canada, 2014.

[2] Coleman-Jensen A., Rabbitt M.P., Gregory C.A., Singh A. (2016). Household Food Security in the United States in 2015.

[3] Bickel G., Nord M., Price C., Hamilton W., Cook J. (2000). Guide to Measuring Household Food Security, Revised 2000.

[4] Ke J., Ford-Jones E.L. (2015). Food insecurity and hunger: A review of the effects on children’s health and behaviour. Paediatr. Child Health.

[5] Tarasuk V., Cheng J., de Oliveira C., Dachner N., Gundersen C., Kurdyak P. (2015). Association between household food insecurity and annual health care costs. Can. Med. Assoc. J..

[6] Tarasuk V., Cheng J., Gundersen C., de Oliveira C., Kurdyak P. (2018). The Relation between Food Insecurity and Mental Health Care Service Utilization in Ontario. Can. J. Psychiatry.

[7] Gundersen C., Tarasuk V., Cheng J., de Oliveira C., Kurdyak P. (2018). Food insecurity status and mortality among adults in Ontario, Canada. PLoS ONE.

[8] Che J., Chen J. (2001). Food insecurity in Canadian households. Health Rep..

[9] Muldoon K.A., Duff P.K., Fielden S., Anema A. (2013). Food insufficiency is associated with psychiatric morbidity in a nationally representative study of mental illness among food insecure Canadians. Soc. Psychiatry Psychiatr. Epidemiol..

[10] Jessiman-Perreault G., McIntyre L. (2017). The household food insecurity gradient and potential reductions in adverse population mental health outcomes in Canadian adults. SSM Popul. Health.

[11] Althoff R.R., Ametti M., Bertmann F. (2016). The role of food insecurity in developmental psychopathology. Prev. Med..

[12] Ashiabi G.S., O’Neal K.K. (2008). A Framework for Understanding the Association Between Food Insecurity and Children’s Developmental Outcomes. Child Dev. Perspect..

[13] Cook J. Impacts of child food insecurity and hunger on health and development in children. Proceedings of the Workshop on Research Gaps and Opportunities on the Causes and Consequences of Child Hunger.

[14] Kirk S.F., Kuhle S., McIsaac J.L., Williams P.L., Rossiter M., Ohinmaa A., Veugelers P.J. (2015). Food security status among grade 5 students in Nova Scotia, Canada and its association with health outcomes. Public Health Nutr..

[15] Jyoti D.F., Frongillo E.A., Jones S.J. (2005). Food Insecurity Affects School Children’s Academic Performance, Weight Gain, and Social Skills. J. Nutr..

[16] Molcho M., Gabhainn S., Kelly C., Friel S., Kelleher C. (2007). Food poverty and health among schoolchildren in Ireland: Findings from the Health Behaviour in School-aged Children (HBSC) study. Public Health Nutr..

[17] Cook J.T., Frank D.A. (2008). Food Security, Poverty and Human Development in the United States. Ann. New York Acad. Sci..

[18] Fram M., Frongillo E., Jones S., Williams R., Burke M. (2011). Children Are Aware of Food Insecurity and Take Responsibility for Managing Food. J. Nutr..

[19] Tarasuk V., Mitchell A., Dachner N. (2013). Household Food Insecurity in Canada, 2011. Research to Identify Policy Options to Reduce Food Insecurity.

[20] Bhattacharya J., Currie J., Haider S. (2004). Poverty, food insecurity, and nutritional outcomes in children and adults. J. Health Econ..

[21] Merriam-Webster Self-Esteem. https://www.merriam-webster.com/dictionary/self-esteem.

[22] Rosenberg M., Schooler C., Schoenbach C., Rosenberg F. (1995). Global self-esteem and specific self-esteem: Different concepts, different outcomes. Am. Sociol. Rev..

[23] Bandura A. (1997). Self-Efficacy: The Exercise of Control.

[24] Richert J., Reuter T., Wiedemann A.U., Lippke S., Ziegelmann J., Schwarzer R. (2010). Differential effects of planning and self-efficacy on fruit and vegetable consumption. Appetite.

[25] Chu Y.L., Fung C., Kuhle S., Storey K.E., Veugelers P.J., Farmer A. (2014). Involvement in home meal preparation is associated with food preference and self-efficacy among Canadian children. Public Health Nutr..

[26] Bartfield J.K., Ojehomon N., Huskey K.W., Davis R.B., Wee C.C. (2010). Preferences and self-efficacy for diet modification among primary care patients. Obesity.

[27] Kamimura A., Jess A., Trinh H.N., Aguilera G., Nourian M.M., Assasnik N., Ashby J. (2017). Food Insecurity Associated with Self-Efficacy and Acculturation. Popul. Health Manag..

[28] Vijayaraghavan M., Jacobs E.A., Seligman H., Fernandez A. (2011). The association between housing instability, food insecurity, and diabetes self-efficacy in low-income adults. J. Health Care Poor Underserved.

[29] Committee on World Food Security Coming to terms with terminology: Food security, nutrition security, food security and nutrition, food and nutrition security. Proceedings of the Thirty-Ninth Session of the EuFMD Commission.

[30] Martin K.S., Colantonio A.G., Picho K., Boyle K.E. (2016). Self-efficacy is associated with increased food security in novel food pantry program. SSM Popul. Health.

[31] Masselink M., Van Roekel E., Oldehinkel A.J. (2018). Self-esteem in Early Adolescence as Predictor of Depressive Symptoms in Late Adolescence and Early Adulthood: The Mediating Role of Motivational and Social Factors. J. Youth Adolesc..

[32] Mulligan A. The Relationship between Self-Esteem and Mental Health Outcomes in Children and Youth. file:///D:/sgodric0/Downloads/eis_self_esteem_mh.pdf.

[33] Bandura A., Pastorelli C., Barbaranelli C., Caprara G.V. (1999). Self-Efficacy Pathways to Childhood Depression. J. Personal. Soc. Psychol..

[34] Hromi-Fiedler A., Bermúdez-Millán A., Segura-Pérez S., Pérez-Escamilla R. (2011). Household food insecurity is associated with depressive symptoms among low-income pregnant Latinas. Matern. Child Nutr..

[35] Chilton M., Knowles M., Bloom S.L. (2017). The Intergenerational Circumstances of Household Food Insecurity and Adversity. J. Hunger Environ. Nutr..

[36] PROOF Latest Household Food Insecurity Data Now. https://proof.utoronto.ca/new-data-available/.

[37] Finance and Treasury Board Economics and Statistics. https://www.novascotia.ca/finance/statistics/archive_news.asp?id=13441&dg=&df=&dto=0&dti=3.

[38] Varni J., Seid M., Rode C.A. (1999). The PedsQL: Measurement model for the pediatric quality of life inventory. Med. Care.

[39] Maximova K., Khan M.K., Austin S.B., Kirk S.F., Veugelers P.J. (2015). The role of underestimating body size for self-esteem and self-efficacy among grade five children in Canada. Ann. Epidemiol..

[40] BMI-for-Age (5–19 Years). https://www.who.int/growthref/who2007_bmi_for_age/en/.

[41] Fung C., McIsaac J.D., Kuhle S., Kirk S.F.L., Veugelers P.J. (2013). The impact of a population-level school food and nutrition policy on dietary intake and body weights of Canadian children. Prev. Med..

[42] Hesketh K., Wake M., Waters E. (2004). Body mass and parent-reported self-esteem in elementary school children: Evidence for a causal relationship. Int. J. Obes..

[43] Veugelers P., Fitzgerald A. (2005). Effectiveness of School Programs in Preventing Childhood Obesity: A Mulltilevel Comparison. Am. J. Public Health.

[44] Laraia B.A., Siega-Riz A.M., Gundersen C., Dole N. (2006). Psychosocial factors and socioeconomic indicators are associated with household food insecurity among pregnant women. J. Nutr..

[45] Rychetnik L., Webb K., Story L., Katz T. (2003). Food Security Options Paper: A Planning Framework and Menu of Options for Policy and Practice Interventions.

[46] Vozoris N.T., Tarasuk V.S. (2003). Household Food Insufficiency is Associated with Poorer Health. J. Nutr..

[47] Melchior M., Caspi A., Howard L.M., Ambler A.P., Bolton H., Mountain N., Moffitt T.E. (2009). Mental Health Context of Food Insecurity: A Representative Cohort of Families With Young Children. Pediatrics.

[48] Emery J.C.H., Fleisch V.C., McIntyre L. (2013). How a Guaranteed Annual Income Could Put Food Banks Out of Business. SPP Research Paper.

[49] Tarasuk V. (2017). Implications of a Basic Income Guarantee for Household Food Insecurity.

[50] Faught E.L., Ekwaru J.P., Gleddie D., Storey K.E., Asbridge M., Veugelers P.J. (2017). The combined impact of diet, physical activity, sleep and screen time on academic achievement: A prospective study of elementary school students in Nova Scotia, Canada. Int. J. Behav. Nutr. Phys. Act..

[51] Loewen O.K., Ekwaru J.P., Faught E.L., Maximova K., Ohinmaa A., Veugelers P. Adherence to recommendations for lifestyle behaviours in childhood and mental health in subsequent years: A prospective study of Canadian children. Proceedings of the International Society for Behavioural Nutrition and Physical Activity Conference 2018.

[52] Garg A., Toy S., Tripodis Y., Cook J., Cordella N. (2015). Influence of Maternal Depression on Household Food Insecurity for Low-Income Families. Acad. Pediatr..

[53] Orth U., Robins R.W. (2013). Understanding the Link Between Low Self-Esteem and Depression. Assoc. Psychol. Sci..

[54] Maynard M., Andrade L., Packull-McCormick S., Perlman C.M., Leos-Toro C., Kirkpatrick S.I. (2018). Food Insecurity and Mental Health among Females in High-Income Countries. Int. J. Environ. Res. Public Health.

[55] Burke M., Martini L.H., Cayir E., Hartline-Grafton H.L., Meade R.L. (2016). Severity of household food insecurity is positively associated with mental disorders among children and adolescents in the United States. J. Nutr..

[56] PROOF Household Food Insecurity in Canada: A Guide to Measurement and Interpretation. https://proof.utoronto.ca/wp-content/uploads/2018/11/Household-Food-Insecurity-in-Canada-A-Guide-to-Measurement-and-Interpretation.pdf.

[57] Blumberg S.J., Bialostosky K., Hamilton W.L., Briefel R.R. (1999). The effectiveness of a short form of the Household Food Security Scale. Am. J. Public Health.

