# Supplementary material for: Canadian Children from Food Insecure Households Experience Low Self-Esteem and Self-Efficacy for Healthy Lifestyle Choices
Source: Nutrients. 2019 Mar 21;11(3):675. doi: 10.3390/nu11030675 (PMC6472195; doi:10.3390/nu11030675)
Supplement: Supplementary file 1 [file nutrients-11-00675-s001.pdf]

**Table S1.** Relationship between food insecurity and self-efficacy for physical activity and healthy eating among grade 5 students (aged 10-11 years) in Nova Scotia, Canada

| **PA Self-efficacy** | | | | | | |
|---|---|---|---|---|---|---|
| | All students | | Girls | | Boys | |
| | B (95%CI) | *p*-Value | B (95%CI) | *p*-Value | B (95%CI) | *p*-Value |
| | **Univariate** | | | | | |
| Food secure | 0.00 | - | 0.00 | - | 0.00 | - |
| Marginal FI[1] | -0.18 (-1.91, 1.55) | 0.838 | -0.31 (-2.71, 2.09) | 0.797 | 0.18 (-2.11, 2.47) | 0.876 |
| Moderate FI[1] | **-2.29 (-3.81, -0.77)** | **0.003** | **-2.80 (-4.66, -0.94)** | **0.003** | -1.69 (-3.94, 0.56) | 0.141 |
| Severe FI[1] | **-2.52 (-4.51, -0.54)** | **0.013** | **-3.22 (-5.71, -0.73)** | **0.011** | -1.57 (-4.67, 1.53) | 0.320 |
| | **Model 1** | | | | | |
| Food secure | 0.00 | - | 0.00 | - | 0.00 | - |
| Marginal FI[1] | 0.33 (-1.39, 2.06) | 0.704 | 0.13 (-2.28, 2.54) | 0.915 | 0.42 (-1.82, 2.65) | 0.709 |
| Moderate FI[1] | **-1.82 (-3.27, -0.37)** | **0.014** | **-2.35 (-4.17, -0.54)** | **0.011** | -1.23 (-3.44, 0.99) | 0.276 |
| Severe FI[1] | **-2.09 (-4.04, -0.14)** | **0.036** | **-2.81 (-5.25, -0.37)** | **0.024** | -1.19 (-4.29, 1.90) | 0.450 |
| | **Model 2** | | | | | |
| Food secure | 0.00 | - | 0.00 | - | 0.00 | - |
| Marginal FI[1] | 0.76 (-0.97, 2.49) | 0.387 | 0.43(-2.01, 2.87) | 0.729 | 0.88 (-1.37, 3.13) | 0.441 |
| Moderate FI[1] | -1.13 (-2.57, 0.31) | 0.125 | **-1.91 (-3.74, -0.08)** | **0.041** | -0.20 (-2.42, 2.02) | 0.861 |
| Severe FI[1] | -1.23 (-3.19, 0.72) | 0.216 | -2.24 (-4.65, 0.16) | 0.067 | 0.01 (-3.13, 3.15) | 0.955 |
| | **Model 3** | | | | | |
| Food secure | 0.00 | - | 0.00 | . | 0.00 | . |
| Marginal FI[1] | 1.44 (-0.28, 3.17) | 0.100 | 1.19 (-1.22, 3.59) | 0.333 | 1.61 (-0.67, 3.90) | 0.166 |
| Moderate FI[1] | -0.27 (-1.82, 1.29) | 0.735 | -1.08 (-3.04, 0.88) | 0.278 | 0.66 (-1.72, 3.05) | 0.586 |
| Severe FI[1] | -0.01 (-2.17, 2.16) | 0.944 | -1.18 (-3.79, 1.43) | 0.373 | 1.39 (-2.05, 4.83) | 0.426 |
| **HE Self-efficacy** | | | | | | |
| | **Univariate** | | | | | |
| Food secure | 0.00 | - | 0.00 | - | 0.00 | - |
| Marginal FI[1] | -1.72 (-3.72, 0.28) | 0.091 | -1.52 (-4.14, 1.10) | 0.255 | -2.53 (-5.32, 0.26) | 0.075 |
| Moderate FI[1] | **-3.08 (-4.81, -1.35)** | **0.001** | **-3.46 (-5.65, -1.26)** | **0.002** | -2.76 (-5.55, 0.028) | 0.052 |
| Severe FI[1] | -2.06 (-4.13, 0.02) | 0.052 | **-3.13 (-5.72, -0.54)** | **0.018** | -1.08 (-4.22, 2.07) | 0.502 |
| | **Model 1** | | | | | |
| Food secure | 0.00 | - | 0.00 | - | 0.00 | - |
| Marginal FI[1] | -1.75 (-3.75, 0.26) | 0.087 | -1.31 (-3.93, 1.32) | 0.329 | -2.39 (-5.15, 0.38) | 0.090 |
| Moderate FI[1] | **-2.88 (-4.59, -1.16)** | **0.001** | **-3.19 (-5.44, -0.94)** | **0.006** | -2.49 (-5.28, 0.29) | 0.079 |
| Severe FI[1] | -1.96 (-4.04, 0.13) | 0.066 | **-2.85 (-5.42, -0.27)** | **0.030** | -0.84 (-3.99, 2.32) | 0.602 |
| | **Model 2** | | | | | |
| Food secure | 0.00 | - | 0.00 | - | 0.00 | - |
| Marginal FI[1] | -1.18 (-3.17, 0.82) | 0.247 | -0.92 (-3.50, 1.65) | 0.481 | -1.63 (-4.40, 1.14) | 0.248 |
| Moderate FI[1] | **-1.94 (-3.68, -0.20)** | **0.029** | **-2.75 (-4.98, -0.52)** | **0.016** | -1.03 (-3.89, 1.82) | 0.477 |
| Severe FI[1] | -0.80 (-2.95, 1.33) | 0.458 | -2.23 (-4.88, 0.42) | 0.099 | 0.93 (-2.22, 4.07) | 0.562 |
| | **Model 3** | | | | | |
| Food secure | 0.00 | - | 0.00 | - | 0.00 | - |
| Marginal FI[1] | -0.72 (-2.72,1.27) | 0.477 | -0.46 (-3.04, 2.13) | 0.729 | -1.11 (-3.95, 1.73) | 0.442 |
| Moderate FI[1] | -1.40 (-3.18, 0.38) | 0.122 | **-2.37 (-4.66, -0.09)** | **0.042** | -0.38 (-3.36, 2.60) | 0.802 |

| | | | | | | |
|---|---|---|---|---|---|---|
| Severe FI[1] | -0.14 (-3.18, 0.38) | 0.906 | -1.87 (-4.82, 1.08) | 0.213 | 1.85 (-1.44, 5.14) | 0.270 |

B: regression coefficient; 95% CI; 95% confidence interval; Model 1 is adjusted for region of residence, body weight status, and gender (in non-gender-stratified models). Model 2 is further adjusted for parental education and Model 3 is further adjusted for household income. Estimates are weighted to represent grade five students in Nova Scotia. Results in bold are statistically significant ($p<0.05$); [1]Food insecurity. PA: Physical activity; HE: Healthy eating
